# Environmental Sources of Bacteria Differentially Influence Host-Associated Microbial Dynamics

**DOI:** 10.1128/mSystems.00052-18

**Published:** 2018-05-29

**Authors:** Cesar Cardona, Simon Lax, Peter Larsen, Brent Stephens, Jarrad Hampton-Marcell, Christian F. Edwardson, Chris Henry, Bill Van Bonn, Jack A. Gilbert

**Affiliations:** a Graduate Program in Biophysical Sciences, University of Chicago, Chicago, Illinois, USA; b Department of Ecology and Evolution, University of Chicago, Chicago, Illinois, USA; c Mathematics and Computer Science, Argonne National Laboratory, Argonne, Illinois, USA; d Department of Civil, Architectural and Environmental Engineering, Illinois Institute of Technology, Chicago, Illinois, USA; e Biosciences Division, Argonne National Laboratory, Argonne, Illinois, USA; f Department of Biological Sciences, University of Illinois at Chicago, Chicago, Illinois, USA; g Aquarium Microbiome Project, A. Watson Armour III Center for Animal Health and Welfare, John G. Shedd Aquarium, Chicago, Illinois, USA; h Department of Surgery, University of Chicago, Chicago, Illinois, USA; University of California, Berkeley

**Keywords:** dolphin microbiome, environmental interactions, host microbiome, microbial ecology, microbial stability, probiotics

## Abstract

Host-associated microbial dynamics are influenced by dietary and immune factors, but how exogenous microbial exposure shapes host-microbe dynamics remains poorly characterized. To investigate this phenomenon, we characterized the skin, rectum, and respiratory tract-associated microbiota in four aquarium-housed dolphins daily over a period of 6 weeks, including administration of a probiotic during weeks 4 to 6. The environmental bacterial sources were also characterized, including the animals’ human handlers, the aquarium air and water, and the dolphins’ food supply. Continuous microbial exposure occurred between all sites, yet each environment maintained a characteristic microbiota, suggesting that the majority of exposure events do not result in colonization. Small changes in water physicochemistry had a significant but weak correlation with change in dolphin-associated bacterial richness but had no influence on phylogenetic diversity. Food and air microbiota were the richest and had the largest conditional influence on other microbiota in the absence of probiotics, but during probiotic administration, food alone had the largest influence on the stability of the dolphin microbiota. Our results suggest that respiratory tract and gastrointestinal epithelium interactions with air- and food-associated microbes had the biggest influence on host-microbiota dynamics, while other interactions, such as skin transmission, played only a minor role. Finally, direct oral stimulation with a foreign exogenous microbial source can have a profound effect on microbial stability.

**IMPORTANCE** These results provide valuable insights into the ecological influence of exogenous microbial exposure, as well as laying the foundation for improving aquarium management practices. By comparing data for dolphins from aquaria that use natural versus artificial seawater, we demonstrate the potential influence of aquarium water disinfection procedures on dolphin microbial dynamics.

## Abstract (precis)

These results provide valuable insights into the ecological influence of exogenous microbial exposure, as well as laying the foundation for improving aquarium management practices. By comparing data for dolphins from aquaria that use natural versus artificial seawater, we demonstrate the potential influence of aquarium water disinfection procedures on dolphin microbial dynamics.

## INTRODUCTION

The relationship between a host and its resident microbiome has been implicated in health, with the microbiota providing benefits to the host through innate immunity, nutrition, and metabolism (1). The microbiome of each individual host is significantly different, while the microbial community composition of an individual host is remarkably stable over time (2). However, despite compositional stability, the relative proportion of each microorganism (what we commonly refer to as community structure) in each host is dynamic. The factors that drive these changes have been identified as diet and disease (1, 3–7). The impact of microbial exposure on host health has been well characterized with respect to disease-causing pathogens, and there is increasing evidence that microbial exposure influences host health through direct immunological stimulation (8). However, while studies have examined interactions between human microbiota and environmental microbiota, it is virtually impossible to characterize all known sources of exogenous microbes in a population (9, 10), and the impact of dietary changes on microbial dynamics is very difficult to control for (7, 11, 12). As such, the influence of environmental microbiota on the dynamics of host-associated microbiota remains largely unknown.

While the interactions between a human and his or her environment- and lifestyle-derived microbes are hard to control for in longitudinal investigations, animals in managed systems have only a limited number of sources of exogenous microbes and are often provisioned with a highly stable diet (13). The sources for aquarium-housed marine mammals, such as dolphins, are essentially limited to water, food, human handlers, and air, and in addition, they have a very stable diet. These animals, therefore, represent a useful model system in which to examine the influence of exogenous microbial exposure on the dynamics of host-associated microbial communities (14). We hypothesized that the dolphin microbiota would exhibit an equilibrium with the regular environmental microbial exposure and that, if a foreign microbial exposure was administered, this would disturb host-associated microbial dynamics.

Dolphin-microbe interactions have been studied with respect to pathogen surveillance (15, 16), identification of potential probiotic strains (17), identification of novel taxa (18, 19), and characterization of variation in the dolphin microbiota across body sites (19–22). In the wild, dolphins are exposed to a broad diversity of microbes in the water and their food and through physical interactions with other dolphins and animals. In an aquarium setting, this exposure is often markedly different. Importantly, aquarium management practices include using stringent disinfection procedures with the intention of minimizing exposure of resident animals to potential pathogenic microbes (23). We aimed to use aquarium-housed dolphins to determine the influence of host-environment interactions on the stability of the dolphin-associated microbiota. We sampled and characterized the microbiota of four dolphins in an intensively managed aquarium setting daily over 6 weeks at different body sites, including skin, rectum, and respiratory exhalant (chuff). We also concurrently characterized microbiota of the hands and noses of the animals’ human handlers, the oceanarium air and water, and the dolphins’ food supply. In addition, we examined the impact of a direct microbe administration intervention starting in week 3, with dolphins randomized to receive either a single-organism or multiorganism probiotic for 3 weeks. As with previous investigations (9, 10), we aimed to quantify the degree of interaction between the environment and the host microbiome to understand whether this interaction correlated with the perceived stability of the microbial community of each animal. Understanding the sources of bacteria and archaea that can temporarily or permanently alter a host’s microbiota could enable new strategies to improve health care for managed dolphin populations and further serve as a potential model for other animals housed in aquatic systems.

## RESULTS

Samples were collected over 42 consecutive days (24 September 2014 to 4 November 2014) in the Shedd Aquarium oceanarium, an indoor, temperature-controlled exhibit that uses recirculating artificial seawater. Sites on 4 Pacific white-sided dolphins (D1 to D4) were sampled, including skin (periumbilicus), rectum, and respiratory tract (through sampling the forceful exhalation referred to as chuff). The hands and noses of the animals’ human handlers, oceanarium air, oceanarium water, and the dolphins’ food (fish and squid blend) were also sampled. For more details, see Text S1 in the supplemental material. A total of 2,370 samples were processed (1,084 of these were analyzed twice as technical replicates, producing a total of 1,286 pooled samples) using 16S rRNA V4 amplicon sequencing. After quality control (see Materials and Methods), including rarefaction of the samples down to 5,000 reads, the final data set comprised 1,214 samples; these 5,929,516 reads clustered with 97% similarity into 19,536 operational taxonomical units (OTUs). Summaries of the number of samples collected on each date and the dominant taxonomic group in each environment are included in the supplemental material (Table S1 and Fig. S1A and B).

10.1128/mSystems.00052-18.1 TEXT S1  Extended information on Materials and Methods. Download TEXT S1, DOCX file, 0.04 MB. Copyright © 2018 Cardona et al. 2018 Cardona et al. This content is distributed under the terms of the Creative Commons Attribution 4.0 International license.

10.1128/mSystems.00052-18.2 FIG S1  (A) Percentages of reads for all samples with more than 5,000 reads (*n* = 1,241) grouped by sample location. Phyla are sorted by abundance, starting with the most abundant phyla in the bottom of the stack. High microbial diversity was observed across all sources; 25 different phyla are identified with an abundance of at least 0.01% reads (592 reads). All sites contained between 21 and 25 different phyla, with air containing 25, dolphins, food, and water, 23, and human trainers, 21. Only the 11 most abundant phyla and “Other phyla” categories are shown. Observations from this data breakdown are as follows: (1) the dolphin rectum harbored nearly as large amounts of *Firmicutes* and *Fusobacteria* as of *Proteobacteria*, (2) food and human nose had comparable amounts of *Firmicutes*, *Actinobacteria*, and *Proteobacteria*, (3) dolphin chuff harbored significant numbers of organisms of Unknown and *Tenericutes* phyla, greater than any other source, and (4) water contained greater abundances of organisms of *Crenarchaeota* archaea and *Verrucomicrobia* phyla than other sources. (B) Percentages of reads for top 23 most abundant families and “Other families” group found in samples with 5,000 or more reads (*n* = 1,241). Families are sorted by abundance, starting with the most abundant families in the bottom of the stack. A total of 297 families were identified. The most abundant 23 families covered only 50 to 75% of the total reads, reflecting that less abundant families still accounted for large portions of the different microbial communities. Download FIG S1, PDF file, 3.9 MB. Copyright © 2018 Cardona et al. 2018 Cardona et al. This content is distributed under the terms of the Creative Commons Attribution 4.0 International license.

10.1128/mSystems.00052-18.4 TABLE S1  Shedd Aquarium sample inventory; counts per date and location. Download TABLE S1, DOCX file, 0.02 MB. Copyright © 2018 Cardona et al. 2018 Cardona et al. This content is distributed under the terms of the Creative Commons Attribution 4.0 International license.

**FIG 1:**
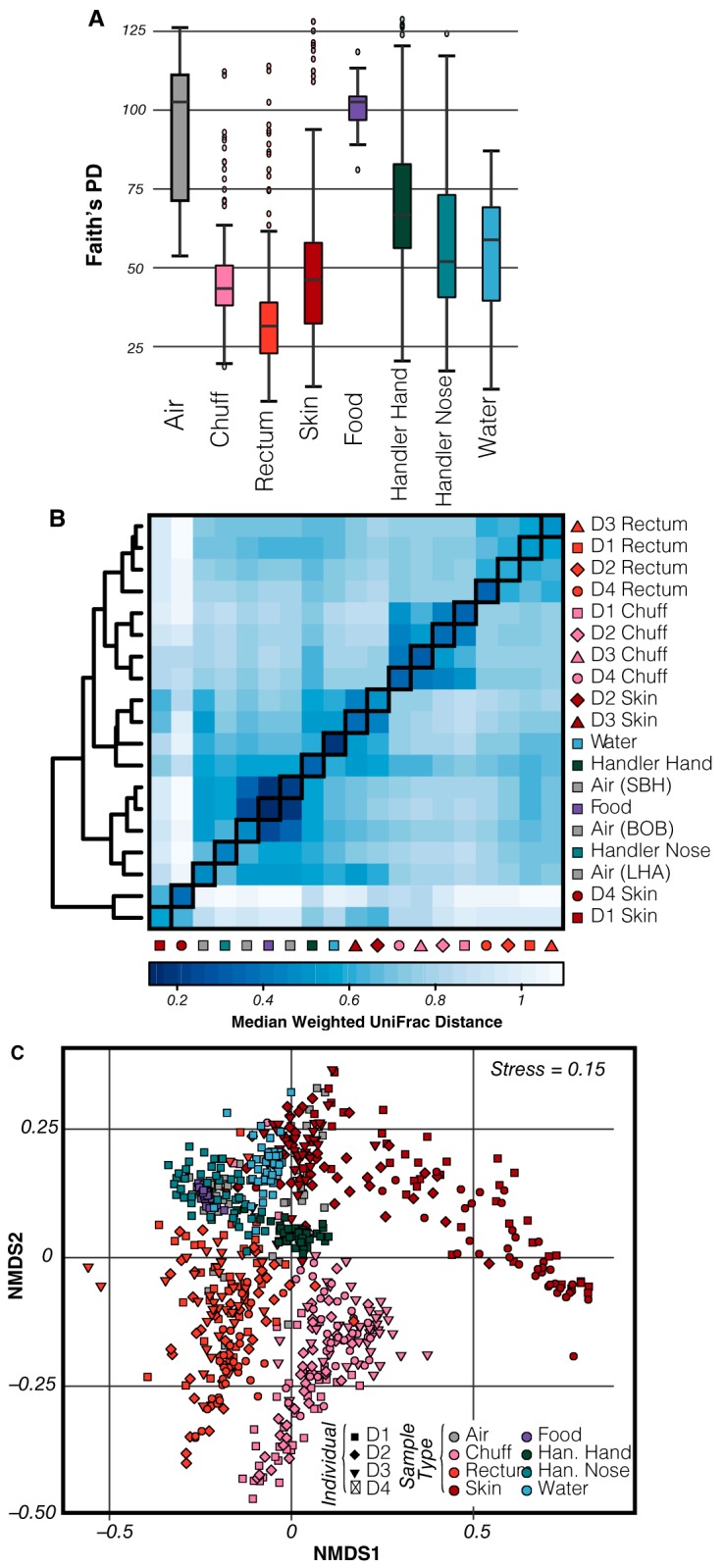
Overview of Shedd Aquarium microbial data. (A) Variations of Faith’s PD alpha diversity, food and air having significantly higher values than other sites and dolphin sites (chuff, rectum, and skin) having lower diversity values. (B) Weighted UniFrac beta diversity hierarchical clustering heat map shows how similar sites tend to cluster together. (C) Weighted UniFrac NMDS plot of Shedd Aquarium samples. Dolphin sites aggregate by sample site rather than per individual, and they are clearly separated from each other. Nondolphin samples are much more intertwined, with air samples showing high similarities to dolphin skin, human hand, and nose, water, and food.

Local diversity within each sample (alpha diversity) and across different samples (beta diversity) was calculated for the 1,214 samples in the study. Alpha diversity (Faith’s phylogenetic diversity [PD]) was variable across sampled sites (Fig. 1A), with food and air having significantly greater alpha diversity (pairwise comparisons using Tukey-Kramer [Nemenyi] test, *P* ≤ 0.002) than all other sites. Overall, as observed in other studies (2, 9, 19) the within-site beta-diversity distances were significantly smaller than the between-site beta-diversity distances (analysis of similarities [ANOSIM], *R* = 0.62, *P* = 0.001), which is exemplified by a weighted UniFrac heat map (Fig. 1B, diagonal versus nondiagonal elements) and nonmetric multidimensional scaling (NMDS) visualization (Fig. 1C). All sites clustered within their environment based on weighted UniFrac distances, except dolphin skin, where the skin of dolphins D1 and D4 had microbial compositions that were significantly different from those of the skin of D2 and D3 (ANOSIM, *R* = 0.45, *P* = 0.001).

At three time points during the study (September 29, October 14, and November 4), more frequent sampling of the dolphin sites was performed (three to six times a day) to build a larger data set focused on diurnal microbiome variations (Table S1). These data showed that the rectum microbiome was the most dynamic microbiome across sampling intervals, with the largest differences for samples taken in the early morning around 0800 and the following sampling times from 1000 to 1800 (permutation *t* test, *P* < 0.001), with *Pasteurellaceae*, *Peptostreptococcaceae*, and *Fusobacteriaceae* OTUs showing sharp increases in abundance in the early morning and a *Brevibacteriaceae* OTU showing a sharp decrease [DESeq2 abs(log_2_FoldChange) > 5 and *P* < 0.001] in the same time frame (Table S2).

10.1128/mSystems.00052-18.5 TABLE S2  Rectum microbiome OTUs that differentially change in abundance when comparing first daily sample around 0800 and subsequent samples from 1000 to 1800. DESeq2 abs(log_2_FoldChange) > 5 and *P* < 0.001. Log_2_FoldChange is the DESeq2 effect size estimate. It tells us how much the abundance seems to have changed among two groups of samples. This value is reported on a logarithmic scale to base 2: for example, a log_2_ fold change of 5 means that the abundance difference for that OTU is changed by a multiplicative factor of 2^5^=32. Download TABLE S2, DOCX file, 0.02 MB. Copyright © 2018 Cardona et al. 2018 Cardona et al. This content is distributed under the terms of the Creative Commons Attribution 4.0 International license.

An inventory of samples from all sites—dolphin, food, air, water, and human—produced a total count of 15,581 (80.2%) OTUs shared between two or more sites, with 2,204 (11.3%) found in all sites sampled (Fig. 1B). Dolphin-associated sites (skin, rectum, and chuff) maintained the greatest proportion of unique OTUs, with 2,543 (13.1%) found only in dolphin samples. In contrast, and perhaps surprisingly, food (which was a stable composite of fish and invertebrates) only had a single OTU that was not shared with another environment. This suggests that, while each environment maintains a core microbiota, likely driven by niche selection, where the environment selects for specific taxa, there are a large number of OTUs overlapping between sites. Common OTUs could be the result of independent selection or OTU transfer between sites. Due to the well-mixed aquatic medium where all dolphins cohabitate, there is indeed ample opportunity for bacterial transfer.

**FIG 2:**
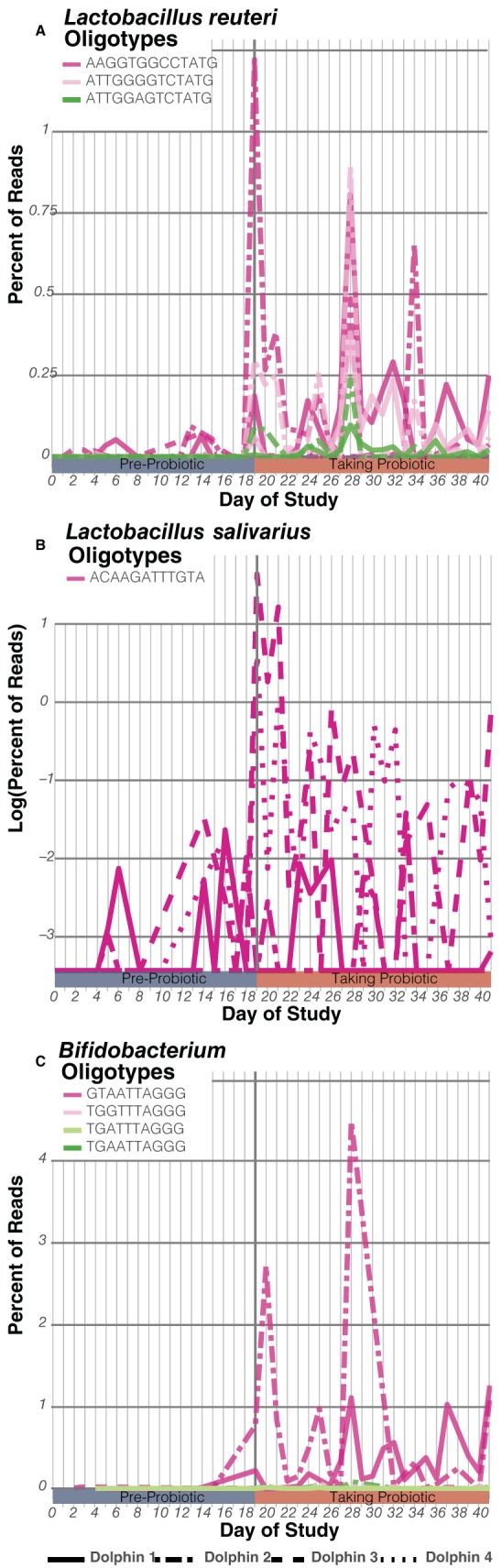
Daily percentages of probiotic oligotype reads found in the dolphin rectum samples. (A, C) Probiotic A oligotypes for Lactobacillus reuteri (A) and *Bifidobacterium* (C) increased significantly for D1 and D2 while taking probiotics. (B) Probiotic B oligotypes for Lactobacillus salivarius (ratio in log scale) increased significantly for D3 and D4 while taking probiotics.

To determine the potential influence of probiotics on the dolphin microbiome, the dolphin population was split into two groups of two, and a different probiotic was administered daily to each group beginning on day 19 of the study. Dolphins D1 and D2 (group A) received a multispecies, Lactobacillus reuteri-dominated consortium (probiotic A), while dolphins D3 and D4 (group B) received Lactobacillus salivarius (probiotic B). For more details, see Text S1. Probiotic A (L. reuteri combination) comprised 9 different OTUs, while probiotic B (L. salivarius) was represented by a single organism. It is important to note that food samples never included the probiotic bacteria supplemented. To track the probiotic organisms in the dolphin population, the probiotic 16S rRNA OTUs annotated as *Lactobacillus* or *Bifidobacterium* were subjected to oligotyping analysis (24). Oligotyping uses entropy to identify unique 16S rRNA V4 marker sequences at sub-OTU resolution. Probiotic A comprised 11 oligotypes, 4 *Lactobacillus* and 7 *Bifidobacterium*; only 3 and 4 of these oligotypes, respectively, were present at ≥5% relative abundance. Probiotic B comprised three *Lactobacillus* oligotypes, one of which accounted for 97% of the reads. The abundances of these oligotypes were quantified in the dolphin rectum samples. The most abundant oligotypes in probiotic A (abundances of ≥5%) were absent or rare (<0.1% reads) prior to probiotic administration and became significantly more abundant (reaching up to 1.2% or 4.4% of total reads for *Lactobacillus* and *Bifidobacterium*, respectively) during administration only in dolphins D1 and D2 (permutation *t* test, *P* ≤ 0.01) (Fig. 2A and C). Similarly, the most abundant oligotype in probiotic B was significantly more abundant following administration only in the dolphins to which it was administered, D3 and D4 (permutation *t* test, *P* < 0.003) (Fig. 2B).

Probiotic administration was associated with a significant decrease in alpha diversity (Faith’s PD) in the rectum-associated microbiome of group A dolphins (comparison of data before versus while taking probiotics; permutation *t* test, *P* = 0.049) but not in other body sites or in group B dolphins (comparison of data before versus while taking probiotics). However, the variance in alpha diversity (Faith’s PD) between samples decreased significantly in both dolphin groups following probiotic administration. This suggests that the microbial community diversity of dolphin-associated sites became more stable following probiotic therapy (Table 1).

**TABLE 1:** Comparison of phylogenetic diversities in dolphin samples before and while taking probiotics

Sampling site	Dolphin group	*P* value for PD by^a^:	
		Permutation *t* test	*F* test
Chuff	A	0.168	**<0.001**
Rectum	A	**0.049**	**<0.001**
Skin	A	0.352	**0.047**
Chuff	B	0.111	**<0.001**
Rectum	B	0.653	**0.019**
Skin	B	0.279	**0.047**

a Permutation *t* test and variance *F* test comparisons for changes in phylogenetic diversity (PD) in different locations and sample subgroups. Significant values (*P* < 0.05) are in boldface.

The alpha diversity for food and water was not significantly affected by whether the dolphins were being administered probiotics, suggesting that the differences observed in the dolphin rectal samples were an influence of dietary probiotics rather than environmental changes. In sites that had a reduced likelihood of being able to influence the dolphin microbiota, for example, air and human hand samples, there were significant changes in alpha diversity and variance during probiotic administration. However, there is no indication that this was associated with probiotic administration to the dolphin diet. In fact, air filters in the oceanarium were changed around the same time that probiotic treatment started, which might explain the sudden drop in air-associated microbial-population diversity (Table S3).

10.1128/mSystems.00052-18.6 TABLE S3  Permutation *t* test and variance *F* test comparisons for changes in phylogenetic diversity (PD) for different animal groups (group A versus group B) and sampling times (samples before versus while taking probiotics) split by different sampling locations. Significant values (*P* < 0.05) are highlighted with pale red. Download TABLE S3, DOCX file, 0.1 MB. Copyright © 2018 Cardona et al. 2018 Cardona et al. This content is distributed under the terms of the Creative Commons Attribution 4.0 International license.

While alpha diversity only changed for one animal group (group A) at a single site (rectum) upon administration of the probiotics, the microbiota compositions of the dolphin sites were significantly different prior to and during probiotic administration and between the two dolphin probiotic groups (groups A and B).

We used ANOSIM to compare whether the distances between samples of the same date period (before and while taking probiotics) were significantly lower than the distances between samples of different date periods. Using ANOSIM with unweighted UniFrac beta diversity distances, the microbiota compositions across all dolphin sites in both periods differed from each other significantly with probiotic administration. In contrast, using weighted UniFrac distances produced significant differences only for dolphin rectum and skin locations. Also, weighted UniFrac produced significant ANOSIM *R* discriminant values (0.081 to 0.122) that were smaller than those produced by unweighted UniFrac. The differing results of the two UniFrac distances suggest that the shifts observed were predominantly due to changes in the proportions of less abundant taxa (~<0.01%) in the dolphin microbiota (Table 2). Also, we tested for compositional differences between microbiota in the dolphin groups (group A versus group B) for both time ranges, before and while taking probiotics, revealing that during both periods, the dolphin groups had significant differences in the microbiota compositions at the sites sampled (ANOSIM, *R* > 0.03, *P* < 0.028), with the exception of dolphin skin prior to probiotic administration. This variance in the dolphin microbiota underlies great individual differences per animal and suggests that our statistical power for investigating community structural shifts related to the probiotic administration was not sufficient with only two dolphins per group (Table S4).

10.1128/mSystems.00052-18.7 TABLE S4  ANOSIM test results for comparison between groups (group A versus group B) and between sampling times (samples before versus while taking probiotics) for different aquarium locations. Weighted UniFrac (WU) and unweighted UniFrac (UWU) distances are shown. Significant values (*P* < 0.05) highlighted with pale red. Download TABLE S4, DOCX file, 0.03 MB. Copyright © 2018 Cardona et al. 2018 Cardona et al. This content is distributed under the terms of the Creative Commons Attribution 4.0 International license.

**TABLE 2:** ANOSIM statistics for differences between before and while taking probiotics for different dolphin sample locations and different groups

Sampling site	Distance^a^	Dolphin group	ANOSIM	
			*R* value	*P* value^b^
Chuff	WU	A	0.007	0.275
Rectum	WU	A	0.122	**0.001**
Skin	WU	A	0.109	**0.001**
Chuff	WU	B	0.010	0.271
Rectum	WU	B	0.081	**0.011**
Skin	WU	B	0.082	**0.004**
Chuff	UWU	A	0.135	**0.001**
Rectum	UWU	A	0.268	**0.001**
Skin	UWU	A	0.273	**0.001**
Chuff	UWU	B	0.215	**0.001**
Rectum	UWU	B	0.185	**0.001**
Skin	UWU	B	0.161	**0.001**

a WU, weighted UniFrac distance; UWU, unweighted UniFrac distance.

b Significant values (*P* < 0.05) are in boldface.

NMDS ordination plots were used to visualize changes in unweighted UniFrac beta diversity distances before and while probiotics were administered (Fig. S2). The skin microbiome showed significant separation of the samples before and after probiotics (group A, ANOSIM, *R* = 0.27, *P* = 0.001; group B, ANOSIM, *R* = 0.16, *P* = 0.001). The rectum microbiome also showed separation of the samples before and after probiotics (group A, ANOSIM, *R* = 0.12, *P* = 0.001; group B, ANOSIM, *R* = 0.27, *P* = 0.001). Interestingly, the group A dolphin rectum samples also showed significant variation in dispersion (permutation test for homogeneity, *P* = 0.001), which could be driving the significant differences found with the ANOSIM test.

10.1128/mSystems.00052-18.3 FIG S2  NMDS ordination presents changes in unweighted UniFrac beta diversity distances before and while taking probiotics for both dolphin groups and all dolphin sites. Download FIG S2, PDF file, 0.4 MB. Copyright © 2018 Cardona et al. 2018 Cardona et al. This content is distributed under the terms of the Creative Commons Attribution 4.0 International license.

**FIG 3:**
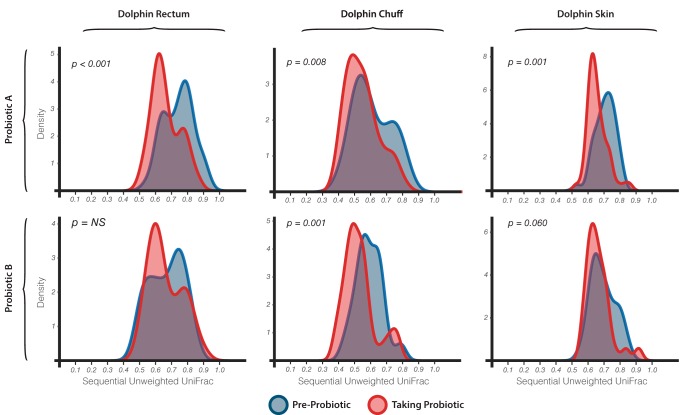
Pairwise UniFrac distances for each consecutive-time pair from the same sample, aggregated and smoothed to distribution functions before and while taking probiotics for different dolphin sites and the two probiotic groups. Permutation *t* tests confirm that the results for dolphin chuff and skin are significantly different for before and while taking probiotics, while the results for dolphin rectum are significantly different only for probiotic A.

To explore the stability in community composition over time within each site (dolphins, water, air, and humans), we calculated pairwise UniFrac distances for each consecutive time pair belonging to the same sample across the entire time series. These distances were used later to determine whether the day-to-day variance in beta diversity was significantly influenced by probiotic administration (Fig. 3). Strikingly, all dolphin sites showed significant differences in average pairwise beta diversity between samples taken pre- and post-probiotic administration (except for dolphin rectum and skin in group B), while all nondolphin samples were not significantly different (except for human nose, *P* ≤ 0.007). The pairwise unweighted UniFrac distances within most dolphin sites during probiotic administration were significantly smaller those than prior to probiotic administration. This suggests that all dolphin microbial communities for group A and chuff microbial communities for group B became significantly more similar day-to-day during probiotic administration, suggesting that the probiotics stabilized the community dynamics. There were no significant differences in any site when comparing the weighted UniFrac distance metrics, again suggesting that any stabilizing effect may have come from changes in the composition of relatively low abundance bacterial taxa.

**FIG 4:**
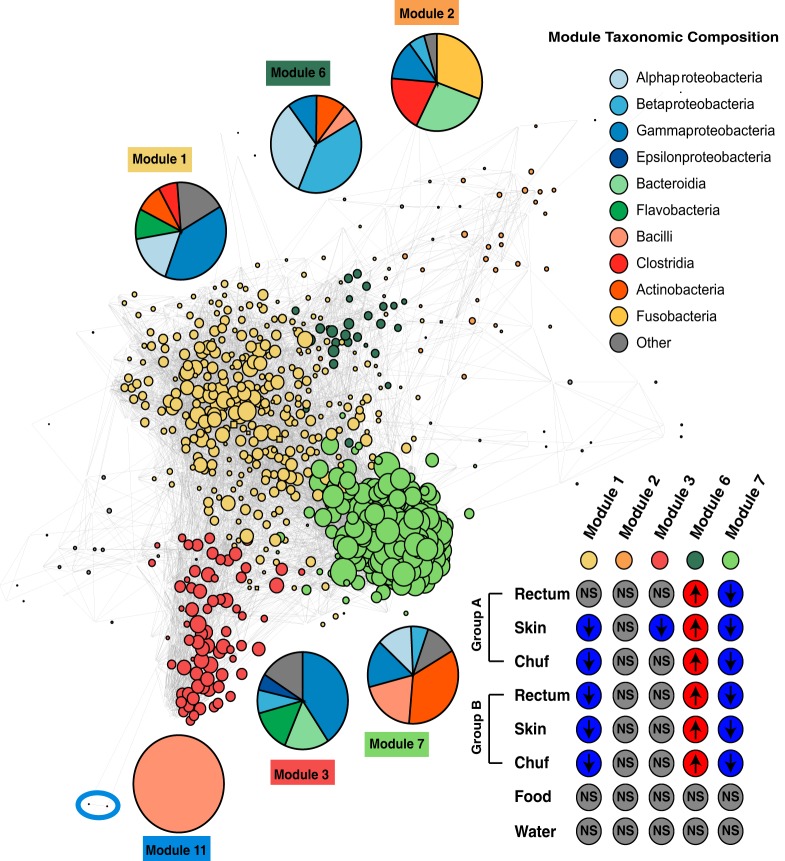
Cooccurrence network for dolphin rectum microbiome. OTUs are the solid-colored circles (nodes), which are connected by lines (edges) if significantly cooccurring in the rectum samples. Size of the node is proportional to the number of connected edges. Microbial communities (modules) were identified following the Walktrap algorithm, based on random walks that produce most likely subnetworks. From a total of 12 modules identified, 6 are further described: modules 1, 2, 3, 6, and 7 are the 5 largest ones (more than 20 OTUs), and module 11 is the module where the single OTU found in probiotic B plus one more OTU are found. All OTUs contained in probiotic A (and 330 additional OTUs) are found in module 1. Pie charts display the taxonomy breakdown of each module at the taxonomic level class. Inset heat map shows results for statistical enrichment test after probiotic administration for OTUs present on the 5 largest modules across the different dolphin, water, and food microbiomes.

Potential keystone OTUs and dense OTU modules that may correspond to distinct subsets of communities within the rectum microbiome were inferred by examining the topology of cooccurrence networks (Fig. 4). A keystone node in a microbial cooccurrence network has been defined as one with (i) high degree (number of connections per node), (ii) low betweenness centrality (number of shortest paths between any two nodes in the graph passing through that node), (iii) high closeness centrality (average distance from this node to any other one), and (iv) high transitivity (probability that adjacent nodes are connected) (25). By this definition, two OTUs assigned to the genera *Kineococcus* and *Brevibacterium* in the phylum *Actinobacteria* (OTU identification numbers [IDs] 543684 and 206826, respectively) were identified as potential keystone taxa, with *Brevibacterium*, interestingly, being the genus that already showed a significant fluctuation of abundance between early morning samples and the rest of the day. These two OTUs were in the top 30% of greatest values for the degree, closeness centrality, and transitivity values, as well as in the bottom 30% of values for betweenness centrality. Studies in the human gut environment have also suggested that *Actinobacteria* fit the definition of keystone taxa, since they are relatively rare, have a high degree of ecological connectedness, and are positively correlated with diversity both within and between different individuals (26). One potential explanation for this similarity is that certain dolphin gut-associated *Actinobacteria* may hold a niche similar to their comparative role in the human gut microbiota.

The dolphin rectal cooccurrence network produced a set of 12 modules. The OTUs from probiotic A coaggregated with 330 other OTUs to make module 1, while the OTUs associated with probiotic B aggregated with only a single other OTU to make module 11. Of the 12 inferred modules, 5 were composed of 20 or more OTUs (modules 1, 2, 3, 6, and 7). Only modules 6 and 7 had significantly differentially abundant OTU counts before and during probiotic administration for both dolphin groups, with module 7 also including the two keystone OTU candidates identified earlier (OTU IDs 543684 and 206826). Following probiotic administration, the abundance of module 6 increased significantly, while that of module 7 decreased significantly (permutation *t* test, *P* ≤ 0.04). The abundance of module 1 also decreased but was only significantly different in the group B dolphins (permutation *t* test, *P* < 0.001). Module 11 was very sparse, with detectable values for only a few days in the study. Each module had its own microbial taxonomic signature; for example, module 1, in which probiotic A clustered, comprised 50% gammaproteobacteria, while module 11, with probiotic B, comprised 100% bacilli (Fig. 4).

For water and food samples (which have the most direct possibility of influencing the dolphin rectum microbiota), we tested whether their microbial communities were enriched by the OTUs present in the five largest dolphin rectum modules (*n* ≥ 20; modules 1, 2, 3, 6, and 7), yet no significant difference was observed between the data obtained before and during probiotic administration (Fig. 4, inset). As before, this suggests that the differences in the rectal samples were not correlated with water or food OTU abundance changes but were likely due to probiotic administration. A full comparison with all the sample types and the enrichment of the modules is available in Table S5.

10.1128/mSystems.00052-18.8 TABLE S5  Statistical test of abundance changes for selected OTUs (those making rectum modules 1, 2, 3, 6, and 7) across dolphin group A and B, water, food, air, and human microbiomes. Increasing abundance after probiotics is marked “Up,” and decreasing abundance after probiotics is marked “Down.” Significant values (*P* < 0.05) are highlighted with pale red. Download TABLE S5, DOCX file, 0.02 MB. Copyright © 2018 Cardona et al. 2018 Cardona et al. This content is distributed under the terms of the Creative Commons Attribution 4.0 International license.

The oceanarium water temperature and chemistry were essentially stable, with low variance in parameter values over the course of our study. Despite this stability, even minor variation in temperature and ammonia concentration correlated significantly with changes in the number of species of the water-associated microbiota observed (water temperature correlated positively and ammonia correlated negatively; Kendall false discovery rate [FDR] corrected, *P* < 0.04). Interestingly, the change in water temperature also correlated positively with changes in the numbers of species observed for dolphin skin, chuff, and rectum (Kendall FDR corrected, *P* < 0.001); however, it is not possible to determine whether this association suggests any mechanistic interaction. In addition, ammonia, pH, and alkalinity showed significant correlations with the alpha diversity of dolphin skin-associated microbiota based on total OTU counts (ammonia and pH correlated positively and alkalinity negatively; Kendall FDR corrected, *P* < 0.001); however, there were no significant correlations between the water chemistry and the Faith’s PD value of the dolphin microbiota. This suggests that, even though the number of species in a community might have changed, the phylogenetic diversity of these communities remained conserved and that the changes observed might be due to fluctuations in rare taxa.

Multiple nonparametric tests of mean similarities were calculated, contrasting samples before and during probiotic administration for each of the water properties measured to rule out any interconnections between the water properties measured and the dates when the probiotic treatment was administered. The correlation of water temperature, alkalinity, and salinity showed a very small but significant reduction in the period during which the dolphins received probiotics (permutation *t* test, FDR corrected, *P* ≤ 0.01). The mean temperatures changed from 16.3 to 15.2°C, alkalinity from 283 to 274 parts per million (ppm), and salinity from 30.8 to 30.7 parts per thousand (ppt) during the probiotic treatment phase. Nitrate and pH increased significantly between these two periods, from a mean of 285 to 381 ppm and from 7.8 to 7.9, respectively (permutation *t* test, FDR corrected, *P* ≤ 0.01). Meanwhile, the chlorine, ammonia, and nitrite values were not significantly different between the two periods of the study.

**FIG 5:**
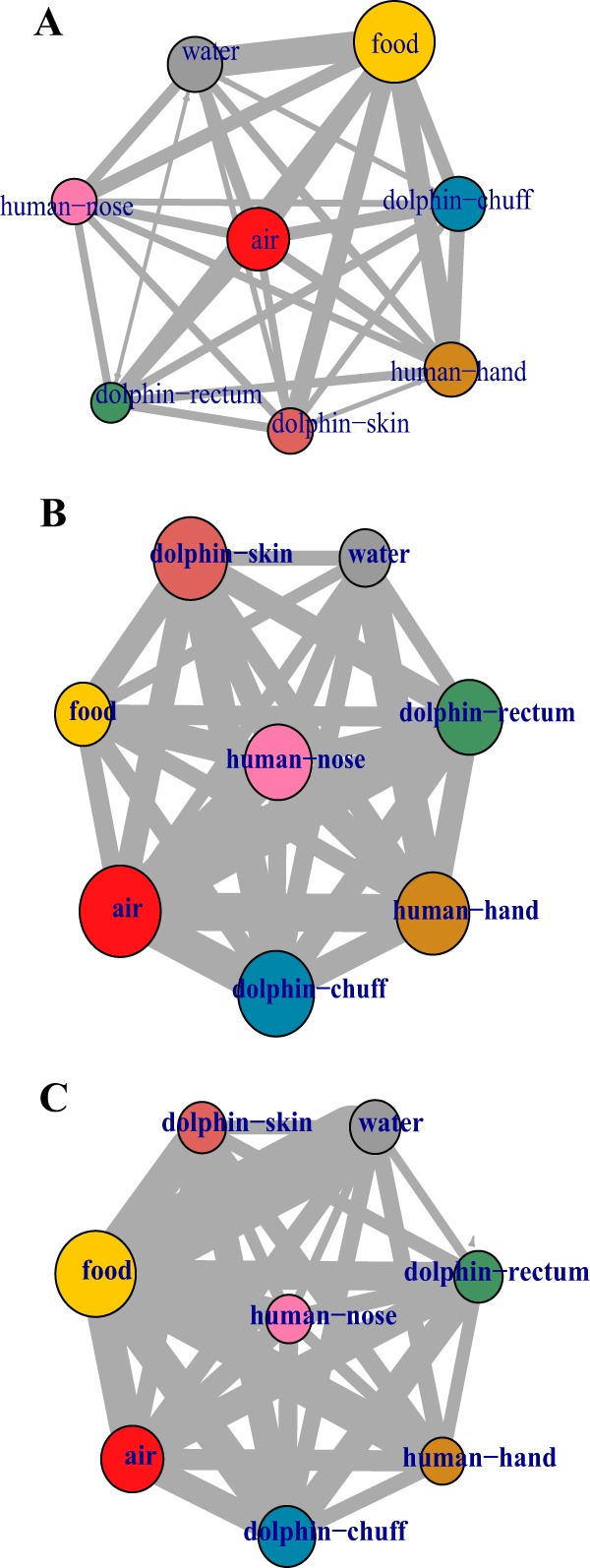
Dynamic Bayesian inference network. Nodes represent the sampled sites, and edge thicknesses the numbers of OTUs that are potentially influencing each other across different sites. (A) Overall site-level summary of the dynamic Bayesian network for the Shedd Aquarium microbiome, where food is the statistically most influential site in the network (binomial, *P* < 0.001). (B, C) Networks before (B) and while taking (C) probiotics.

Potential temporospatial correlation between OTUs from different sites (dolphin, water, food, air, and human) was assessed using dynamic Bayesian network (DBN) analysis. DBNs relate OTU counts over adjacent time steps, providing a metric for the influence of specific OTU abundances at time *t* to various other OTU abundances observed at time *t* + 1. For simplicity of visualization, the network was summarized at the site level, showing a multidirectional exchange among all sites (Fig. 5A). Site-specific OTUs at time *t* that significantly correlated with OTUs from other sites at time *t* + 1 were identified with binomial tests, and food-associated OTUs had the greatest significant association with OTUs in other sites and had influence that was significantly greater than random (*P* < 0.001). In addition, the OTUs associated with the microbiota of dolphin rectum were also significantly associated with the microbiota of dolphin skin (*P* ≤ 0.002), which could be seen either as a mechanistic effect (27) or a direct physical exposure, as dolphin feces are readily mixed into the water and could, therefore, influence the dolphin’s umbilicus region, which was the site sampled for skin microbiota.

To determine the intersection between environmental and probiotic influence, we constructed two additional DBNs, one before and one during probiotic administration (Fig. 5B and C). The before-probiotics network inferred that the abundances of air (*P* < 0.001) and food (*P* = 0.002) OTUs conditionally predicted the abundances of OTUs on other surfaces. Therefore, changes in the abundances of air- and food-associated taxa correlated more frequently with the changing abundances of the microbiota of other surfaces, which suggests that air and food have the biggest impacts on the dolphin-associated microbiota. For the during-probiotics network, the number of OTU abundances conditionally inferred from food OTUs nearly doubled, making food the single site in the network with significant influence on taxon abundances on other surfaces (*P* < 0.001), possibly because the probiotics were interacting with the food microbiome.

**FIG 6:**
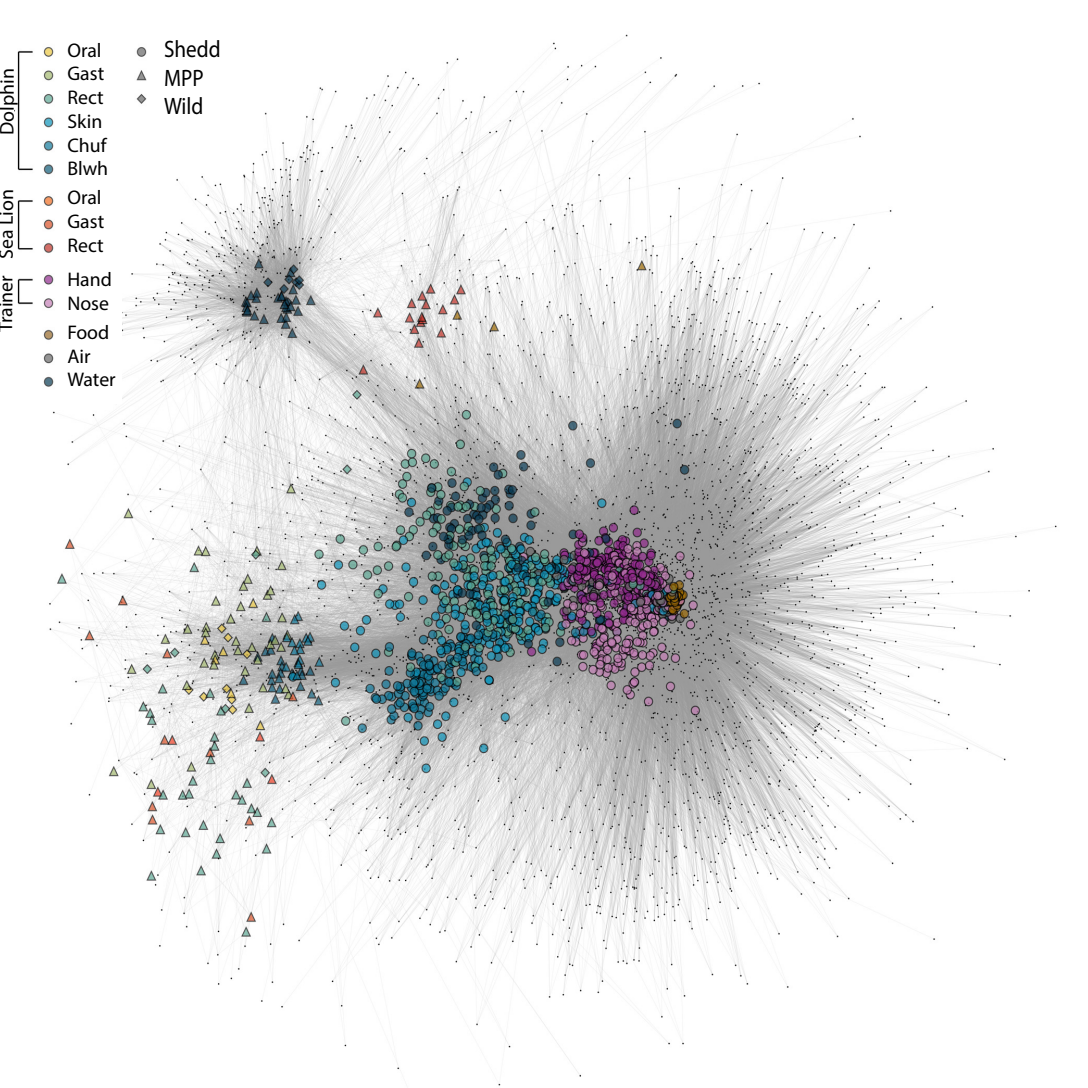
Bipartite network, from unweighted UniFrac distances, features Shedd, MMP, and Sarasota microbiome comparisons. Human samples were only taken in the Shedd Aquarium. Sea lion, dolphin blowhole, and dolphin gastric samples were only taken in the MMP.

A recent study (19) examined the microbiota of dolphins and sea lions at San Diego Bay, California, and a second, undisclosed location, both part of the Navy Marine Mammal Program (MMP) (28, 29), as well as the microbiota of wild dolphins from Sarasota Bay in Florida that were sampled as part of a catch and release conservation program (30, 31). We combined these data sets with data from the current study (Table S6) and created a bipartite network (which includes both samples and OTUs) displaying sample similarity as a function of how close samples are to each other (Fig. 6). Statistical calculations determined that the greatest difference among locations was in the unweighted UniFrac beta diversity of the water (ANOSIM, *R* = 0.72, *P* = 0.001). Seawater samples from the MMP sites and Sarasota had greater alpha diversity (observed phylum-level bacterial diversity) than the corresponding animal and food samples from these sites; in contrast, the artificial seawater at the Shedd Aquarium had lower phylum-level diversity than the corresponding dolphin and food microbiota from the current study (Fig. S1A).

10.1128/mSystems.00052-18.9 TABLE S6  Samples from Bik et al. (19) (MMP and wild) and Shedd Aquarium studies, including close reference OTUs and samples rarefied to a depth of 1,000 reads only. Download TABLE S6, DOCX file, 0.02 MB. Copyright © 2018 Cardona et al. 2018 Cardona et al. This content is distributed under the terms of the Creative Commons Attribution 4.0 International license.

**FIG 7:**
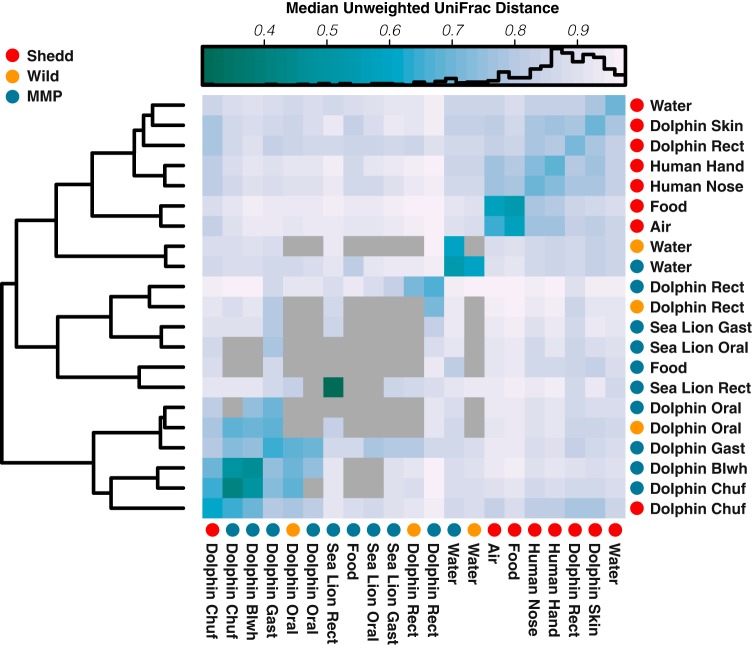
Median unweighted UniFrac distances between three dolphin habitats: Shedd, MMP, and Sarasota. The rows and columns have been grouped with hierarchical clustering to present more similar patterns next to each other. Darker blue squares represent higher similarities between samples. Gray squares represent comparisons with less than the minimum 100 UniFrac distances to confidently compute the statistics.

Beta diversity (unweighted UniFrac) within rectum samples was significantly smaller within locations (MMP, Sarasota, and Shedd) than between locations (ANOSIM, *R* = 0.79, *P* = 0.001). MMP and Sarasota rectum microbiomes were more similar to each other than to those from the Shedd Aquarium (MMP versus Sarasota, ANOSIM, *R* = 0.25, *P* = 0.02; MMP versus Shedd, ANOSIM, *R* = 0.84, *P* = 0.001; and Sarasota versus Shedd, *R* = 0.69, *P* = 0.001), which could have resulted from batch effect, as these samples were generated in a different laboratory, but could also be an effect of exposure to natural seawater versus artificial seawater. In clustering the unweighted UniFrac distances for water, food, air, human handlers, and animal samples for the three locations (MMP, Sarasota, and Shedd), only dolphin chuff samples were aggregated together, regardless of the geographical location (Fig. 7). This points to a very distinctive microbiome in the chuff, which, as shown previously, is also the site that harbored the greatest number of bacteria without a known phylogeny (Fig. S1A) (18, 32).

## DISCUSSION

In this study, we explored the microbiota of Pacific white-sided dolphins over a 6-week period, determined the association between their microbiomes and those of the environments they came into contact with, and observed the impact of probiotic administration on the host microbiota. In the human microbiome, disturbances due to medications, such as antibiotics, potential immune system activation due to pathogenic exposure, or diet changes can lead to sudden and dramatic changes in the structure of the microbiome (12). However, the degree to which the environmental microbiota interact with the host microbiota and host environment remains unknown.

The Shedd Aquarium comprises a closed ecosystem where each environment (human, water, air, and animal) shows characteristic microbiota that are predicted to strongly influence each other, with more than 80% of OTUs shared between two or more sites. This implies that there is a continuous exposure of microbes between sites, which is important to understand when implementing any microbial manipulation of the ecosystem, particularly with water management practices. Previous studies have shown similar patterns. For example, individuals and pets under the same roof still maintained their unique signature microbiome, despite constant changes in the structures of their microbiota (9). Also, despite different environmental sources associated with seawater at different locations, tunicates maintain unique microbial signatures, with some degree of overlap between nearby tunicate populations (33). While the current study did not perform direct manipulation of the aquarium microbiota through controlled intervention, these data still demonstrate that air- and food-associated microbial exposures have the largest potential influence on the host-associated microbial dynamics. When probiotics are added to the food, the microbiota in the mixture becomes the dominant influence on dolphin-associated microbial dynamics.

Despite minimal differences in the temperature, ammonia, salinity, and pH values of the dolphin habitat water day-to-day, the small variations that did occur correlated with changes in the microbial community diversity of the water and dolphin sites. However, this was likely associated with the dynamics of low abundance bacterial taxa. This still suggests that even tight control of environmental variables can elicit shifts in the structure of the microbiota but that such changes do not have dramatic impacts on the phylogenetic diversity of microbial-community composition. Food and air microbiota maintained the greatest alpha diversities, and in the absence of probiotics, they also hosted the greatest numbers of OTUs that conditionally influenced abundances in other microbiomes (binomial, *P* < 0.002). OTUs associated with dolphin rectum significantly influenced the microbiota of the dolphin skin, either by direct transfer (from rectum through water to skin) or through immune modulation (27). The rectum microbiome cooccurrence network also suggests that OTUs associated with the genera *Kineococcus* and *Brevibacterium* could be keystone taxa in the dolphin gut. *Kineococcus* has been associated with human oral microbiota, where it coaggregates to support biofilm formation (34). It is possible that *Kineococcus* is also playing a role in community aggregation for the dolphin rectum microbiome. *Brevibacterium* has been associated with human skin, where it is involved in sulfur metabolism (35); it is possible that it could be playing a similar role in dolphin rectum. Its presence also supports the supposition of continual exposure of microbiota between skin and rectum in this well-mixed aquatic environment. *Brevibacterium* also showed diurnal oscillations, a phenomenon which has proven likely to increase metabolic homeostasis (36) and could potentially be connected to water oxidation-reduction potential (ORP) cycles (see Materials and Methods). Comparison between the Shedd, MMP, and Sarasota environments demonstrated that the dolphin, food, and water microbiota were all unique to the specific locations, although MMP and Sarasota were more similar to each other than either was to Shedd. This suggests that either differences in water and aquarium management or a substantial batch effect in how and where samples were processed influenced the community composition and structure substantially. However, the beta diversities for dolphin chuff were not significantly different between sites, suggesting that this is an extremely conserved environment and, also, that a batch effect may not explain the other dissimilarities. The chuff-associated microbiota also harbored the most bacterial taxa that could not be reliably identified to at least a phylum (Fig. S1A); indeed, previous studies have characterized dolphin and whale chuff microbiota and found similar results (18, 32).

Moreover, the artificial seawater used by the Shedd Aquarium oceanarium had a significantly lower diversity than the seawater at both MMP and Sarasota, and while seawater generally had a greater diversity than host-associated environments, the artificial seawater was less diverse than the dolphin sites in our study. Water management practices can have a profound impact on the microbial population diversity of aquarium water (37), which suggests that if found to be health promoting, it might be possible to promote an increase in the diversity of microbes in artificial seawater, so as to more closely resemble that of the animal’s native seawater. While some authors (38) describe how organic load and maturation of water increase the microbial carrying capacity of aquatic habitats, making them more stable and less open for opportunistic proliferation, other scientists present contrasting results (39), suggesting that increasing microbial exposure will also increase pathogen burden. It is essential that research be done to inform recommendations, and such research must carefully examine the interactions between water chemistry, the microbiome, and animal health indicators. We suggest that optimal management practices will result in environmental microbiomes somewhere in between those realized due to contemporary disinfection practices and those in which no disinfection exists, as in native environments. Probiotic supplementation of mouse and farm animal diets has shown how certain microbial strains are able to provide higher resistance to pathogens, possibly by competitive exclusion or by stimulation of host immune system responses (40–43). In the case of the current study, we had two probiotic formulations, one with a single organism and one with more than 10 organisms. Both probiotics used in this study appeared to be associated with an increase in microbial community stability, but the L. reuteri-dominated multimember consortium led to a greater increase in stability than the single L. salivarius probiotic. Also, the L. reuteri-dominated formulation increased the stability of the microbiota in all three dolphin sites (chuff, rectum, and skin), while the single L. salivarius probiotic only increased stability for dolphin chuff. This evidence supports the findings from prior studies of immune modulation (44) specific to *Lactobacillus* and *Bifidobacterium* (45–48), which have already been identified as likely beneficial for the hosts.

The topology of the cooccurrence network for OTUs in the rectum samples provided an insight into the microbial ecology of the rectum and, therefore, into the dolphin gut microbiota. Strains associated with the L. reuteri-dominated formulation (including *Bifidobacterium*) formed a large cluster with more than 300 other taxa, which suggests that these probiotics show changes in abundance that match this community more than any other members of the gut microbiota. Meanwhile, changes in the abundance of the L. salivarius strain only correlated with a single host-associated OTU. As the L. reuteri formulation is a multispecies probiotic that showed cooccurrence clustering with the largest number of host-associated taxa and was associated with the greatest increase in host-microbiota stability, this might suggest that the stabilization effect of probiotics may be more likely if the formulation can establish interconnections with the existing host microbial community, although of course, other explanations could also be relevant. For example, the L. reuteri-dominated formulation may have exerted the biggest influence on the immune system, which led to the largest number of changes in the abundance of the gut microbiota, and hence, the association density was related to indirect influence. The addition of probiotics to the diet also seems to change the topology of the microbial network of interactions, as it almost doubled the number of OTU abundances conditionally associated with food OTUs, making food the single site in the network with significant influence on the abundances of taxa on other surfaces (binomial, *P* < 0.001).

In summary, the current study demonstrates that probiotic administration was associated with an increase in the stability of host-associated microbiota. The treatment was also associated with changes in the network structure of correlations in microbial abundance, resulting in food microorganisms having a dominant influence on the OTUs associated with dolphin and nondolphin sites. The study suggests that while environmentally derived exogenous bacteria can exert some influence on the dynamics of host microbiota, these differences are not as great as those resulting from direct stimulation with a completely foreign exogenous microbial source. It is important to state that changes in influence and stability statistics were observed using the unweighted UniFrac metrics, suggesting that many of the stability effects are driven by changes in rare species only. This suggests that in host-associated systems, equilibrium is achieved in the presence of common microbial exposures, for example, those in the immediate usual environment. It also suggests that food and air, and hence, oral, gastrointestinal tract, and respiratory tract interactions, have the largest effect overall. Meanwhile, uncommon microbial exposures can have a profound impact on the stability and structure of microbial associations. Demonstrating that direct probiotic administration influences host microbial community dynamics has major implications for animal health and aquarium management practices.

## MATERIALS AND METHODS

### Animals included in the study.

Individual animals sampled included four Pacific white-sided dolphins (Lagenhorynchus obliquidens), including three females that originated from the North Pacific Ocean and had been housed at Shedd for over 20 years and one aquarium-born male. The approximate ages of the females during the study period ranged from 27 to 29 years, and the male was of known age, 2 years, 4 months, to 2 years, 5 months, during the study period. For the purpose of this paper, the labels D1 to D4 each uniquely identify one of the dolphins.

### Animal-trainer interactions.

All dolphins included in the study were housed together in an indoor, closed recirculating synthetic seawater habitat of approximately 11.3-million-liter total volume. The habitat is subdivided into several enclosures by a gate system; however, water circulates freely between all. In addition to the four dolphins studied, four California sea lions (Zalophus californianus) and seven beluga whales (Delphinapterus leucas) occupied adjacent enclosures. The dolphins were fed in simultaneous session, with each animal being fed individually by a dedicated trainer but physically separated from each other to ensure that all food items, including those containing probiotic capsules, were consumed only by the target animal. During the study period, all trainers worked with all animals and rotated between them at different sessions. Trainers were not assigned to specific animals. During the study period, a total of 27 individual trainers were sampled.

The 24-h activity cycle of the oceanarium habitat was regular during the study period. Initial feeding sessions with the animals were conducted between 0800 and 0900 daily. During weekdays, follow-on scheduled sessions occurred with the animals at 1030, 1230, and 1430. On weekend days, the additional scheduled sessions were conducted at 1030, 1230, 1430, and 1600. Unscheduled sessions were conducted between the scheduled sessions so that each animal was interacted with and fed up to a total of eight sessions daily.

### Water ORP.

In many of the aquarium systems that use ozone contact disinfection, which is the only oxidant used in the study system, the oxidation-reduction potential (ORP) demonstrates a daily cycle, gradually increasing overnight and dropping during the morning hours when daily activities begin. This is presumed to be a result of the increased bioload on the system during feeding and cleaning activities. System ORP was not measured during the study period but has shown this pattern when it has been measured in the past.

### Samples collected.

Dolphin sites sampled included skin (periumbilicus), rectum, and respiratory tract (forceful exhalation referred to as chuff). In addition, hands and noses of the animals’ human handlers, oceanarium air, oceanarium water, and the dolphin’s food (fish and squid blend) were sampled. A total of 2,370 samples were processed. For details on the sample collection methodology, see Text S1 in the supplemental material.

### Amplicon library preparation.

Genomic DNA was extracted from environmental samples using the PowerSoil DNA isolation kit (Mo Bio, Inc.), and genomic DNA was amplified using the Earth Microbiome Project (EMP) protocols (http://www.earthmicrobiome.org [49]). For more details, see Text S1.

### Sequence processing and statistical analysis.

A total of 2,370 samples were sequenced on the Illumina MiSeq. After 1,084 technical replicates were identified, the data were reduced to 1,286 pooled samples. The reads were quality filtered using Quantitative Insights Into Microbial Ecology (QIIME) (50), and downstream processing of sequence data utilized QIIME, R, and oligotyping (24). For more details, see Text S1.

### Cooccurrence and inference networks.

The cooccurrence network was calculated for rectum samples only, with a resulting network of 717 nodes and 68,515 edges. In preparation for network creation, we removed OTUs with abundances of less than 0.01% of the total number of OTUs, for a subset of 717 OTUs. Determination of cooccurrence of OTUs used the WGCNA package (51). OTUs from all surfaces were prefiltered with DESeq2 (52) to select only the OTUs with a statistically different abundance in at least one sampled site {41 OTUs; *P* = 0.001 and abs[log_2_(abundance)] > 1}, and DBNs were created via Banjo (53). Network properties and visualization were done with the CAVNet R package (54). For more details, see Text S1.

### Data availability.

Sequenced reads are available in the QIITA database under study identification number (ID) 11279 (https://qiita.ucsd.edu/study/description/11279). For more details, see Text S1.
