# Supplementary material for: Environmental Sources of Bacteria Differentially Influence Host-Associated Microbial Dynamics
Source: mSystems. 2018 May 29;3(3):e00052-18. doi: 10.1128/mSystems.00052-18 (PMC5974334; doi:10.1128/mSystems.00052-18)
Supplement: TEXT S1 [file sys003182234s1.docx]

## SI - Materials and Methods

### Sample Collection

During the study period that extended from 24 September through 4 November 2014 samples were collected daily. Sample sources included water from the dolphin habitat, air samples (passive settling plates), food fish and squid blend, animals (three sites; skin, chuff, and rectum) and animal handlers (two sites; skin of hands and nose). All sample collection was approved by the intramural Shedd Research Committee and was conducted under the University of Chicago IRB approval (protocol # 14-0667).

Water samples consisted of 500 ml of exhibit water collected as a ‘grab sample’ from the exhibit into a sterile Nalgene™ bottle and then passed through a 0.22 µ Millipore Sterivex™ filter. Air samples were collected from three locations within the oceanarium space (BOB = Beluga Overlook Balcony, SBH = Secluded Bay Habitat, LHA = Large Habitat A line) using passive settling plates. Passive air 10 cm x 15 cm aluminum plates were sterilized with isopropyl alcohol prior to every collection period and allowed to settle for 24 hours before sampling. Samples were collected by swabbing the plate with a sterile cotton tipped applicator. Food fish was sampled by homogenizing the representative daily animal ration in a commercial food grade blender, then taking a subsample of the homogenate with a sterile cotton tipped applicator. Animal and handler skin sites and handler nasal samples were collected directly onto sterile cotton tipped applicators passed vigorously back and forth along the sampled surface multiple times for approximately 3 seconds. Animal stool samples were collected by passing a sterile red rubber catheter *per rectum* and directly swabbing feces from the surface of the tube following removal. Animal skin was swabbed from the area around the umbilicus. Animal nasal samples were collected by holding a sterile petri dish over the animals’ external nares (blowhole) and having the animal forcefully exhale (chuff) under behavioral control. The interior surface of the petri dish was then immediately swabbed with a sterile cotton tipped applicator.

At three time points during the study (9/29, 10/14 and 11/04), more frequent sampling of the dolphin sites was performed (three to six times a day) to build a larger dataset focused on diurnal microbiome variations (Table S1). All swabs, filters were immediately transferred to capped sterile 15 ml polypropylene conical VWR® high performance centrifuge tubes or whirlpak™ bags and frozen at - 80° C until DNA extraction.

### Probiotics administration

Mid-way through the study (day 19) all dolphins were started on oral probiotics. The probiotic pills were put into fish fed to the animal. It is important to distinguish that this fish was not part of the food blend described above, therefore the probiotic samples were never captured in the food microbiome sequences. Two dolphins (Group A: D1 and D2) were given 2 daily pills of Lactobacillus reuteri (L. reuteri) combination product (*Lactobacillus acidophilus, Bifidobacterium longum, Lactobacillus reuteri, and Lactobacillus rhamnosus*) (1) that had been used previously by other animals in the collection but never given to them. Two dolphins (Group B: D3 and D4) were given 1 daily pill of *Lactobacillus salivarius (L. salivarius)* (2–4) which had never been used in the study collection previously.

One dolphin in each of the groups had previously received a probiotic. In Group A, dolphin D2 had previously received a product containing *B. coagulans*, *B. subtilis* and *E. faecalis* [Three Lac® Global Health Trax] (last administered 4 March 2013). In Group B, dolphin D4 had previously received L. reuteri (last administered on 11 July 2014). The other dolphin in each group (D1 and D3) had never previously received a probiotic. Samples of the two probiotics were sequenced (as described below) to confirm their formulation (Table S2).

### Amplicon Library Preparation

Genomic DNA was extracted from environmental samples using the PowerSoil DNA Isolation Kit (MO BIO) and genomic DNA was amplified using the Earth Microbiome Project (EMP) protocols [www.earthmicrobiome.org](http://www.earthmicrobiome.org) (5). Using a barcoded primer set adapted for MiSeq by adding nine extra bases in the adapter region of the forward amplification primer that support paired-end sequencing (6) V4 region of the 16S rRNA gene was amplified with primers (515F-806R) that included the Illumina flow cell adapter sequences. The reverse amplification primer also contained a twelve base barcode sequence that supports pooling of up to 2,167 different samples in each lane. Each 25 ul PCR reaction contained 12 ul of MoBio PCR Water (Certified DNA-free), 10 ul of 5 Prime HotMasterMix (1x), 1 ul of Forward Primer (5 uM concentration, 200 pM final), 1 ul Golay Barcode Tagged Reverse Primer (5 uM concentration, 200 pM final), and 1 ul of genomic DNA. The PCR conditions are as follows: 94 °C for 3 minutes to denature the DNA, with 35 cycles at 94 °C for 45 seconds, 50 °C for 60 seconds and 72 °C for 90 seconds, with a final extension of 10 minutes at 72 °C to ensure complete amplification. Following PCR, amplicons were quantified using PicoGreen (Invitrogen) and a fluorescence plate reader. Volumes of each of the products were pooled into a single tube so that each amplicon was represented equally. This pool was then cleaned using the UltraClean® PCR Clean-Up Kit (MoBio), quantified using Qubit (Invitrogen), and diluted to 2nM, denatured, and then diluted to a final concentration of 2 pM with a 30% PhiX spike for loading on the Illumina MiSeq sequencer.

### Sequence Processing and Statistical Analysis

A total of 2,370 samples were processed (1084 of them with a paired technical replicate sample, producing a total of 1,286 pooled samples) were sequenced on the Illumina MiSeq. The reads were quality filter using Quantitative Insights Into Microbial Ecology (QIIME) (7) by (1) exact match to an expected multiplex barcode sequence, (2) zero ambiguous base calls, (3) a minimum Phred score of 20 across the entire length of the read, (4) removing chimeras using UCHIME 6.1(8) and the Greengenes reference database version 13.5 (9) . Sequences were clustered together at 97% identity using the QIIME script *pick_open_reference_otus.py* and the UCLUST algorithm using the same Greengenes database as a reference. Technical replicates were obtained by taking two consecutive swabs at the site of interest and after quality control were later merged. Samples with less than 5,000 high-quality reads were discarded. OTUs comprising of less than 10 reads, present in less than 3 samples or assigned to special Greengenes taxonomies *c__Chloroplast* and *f__mitochondria* were removed. The resulting OTU table contained 1214 samples and 53,136 OTUs and was used for dynamic bayesian analysis networks. For the remaining analyses the dataset was subsampled to 5,000 reads, resulting in 1214 samples with 19,536 OTUs.

To compare the Shedd aquarium and Bik et al. (10) data, we used only OTUs from both datasets matching the reference database Greengenes 13.5 and rarefied to 1,000 reads. Denovo OTUs from our Shedd aquarium data were excluded by using the QIIME script *filter_otus_from_otu_table.py* and OTUs from the Marine mammals paper were picked again using the QIIME script *pick_closed_reference_otus*.py. Strain level analysis was performed used the Oligotyping tool (11), which uses entropy to identify unique 16S rRNA V4 marker sequences at sub-OTU level resolution. Downstream statistical processing of sequence data utilized QIIME and various R packages for data manipulation and statistics: plyr (12), dplyr (13), tidyr (14), vegan (15), PMCMR (16), VennDiagram (17) and Deducer (18).

### Co-occurrence and inference Networks

The co-occurrence network was calculated for rectum samples only, with a resulting network of 717 nodes and 68,515 edges. In preparation for network creation we removed OTUs with abundances less than 0.01% of the total number of OTUs, for a subset of 717 OTUs. Co-occurrence of OTUs was defined based on Pearson correlations using the WGCNA package (19). The nodes in each network represent OTUs and the edges connecting the nodes represent correlations between OTU pairs. We adjusted all P-values for multiple testing using the Benjamini and Hochberg false discovery rate (FDR) controlling procedure (20), as implemented in the multtest R package (21). The direct correlation dependencies were distinguished using the network deconvolution method (22). Edges were pruned to keep only high correlation coefficients and significant FDR-adjusted P-values for correlation. The cutoff of correlation coefficients was determined as 0.81, through random matrix theory-based methods (23) The cutoff of FDR-adjusted P-values was 0.01. Network properties and visualization was done with the CAVNet R package (<https://bitbucket.org/JackGilbertLab/cavnet>) (24)

In building the dynamic bayesian network, OTUs from all surfaces were prefiltered with DESeq2 (25) to select only the 41 OTUs with a statistically differential abundance in at least one sampled site (*P*=0.001 and *abs(log2(abundance))>1*). The actual dynamic bayesian network was created via Banjo (26) with parameters: quantile discretization=5, search algorithm=Simulated annealing, max parents=5, markov lag=1 and report and combine highest-scoring networks=5, for a resulting network of 241 nodes (OTU-location pairs) and 563 edges. Nodes were later collapsed into 8 location nodes for easier visualization. R package sna (27) and CAVNet were used to read banjo networks and calculate network attributes.

# References

1. Forsythe P, Inman MD, Bienenstock J. 2007. Oral Treatment with Live Lactobacillus reuteri Inhibits the Allergic Airway Response in Mice. Am J Respir Crit Care Med 175:561–569.

2. Chaves B d., Brashears M m., Nightingale K k. 2017. Applications and safety considerations of Lactobacillus salivarius as a probiotic in animal and human health. J Appl Microbiol 123:18–28.

3. Feighery LM, Smith P, O’Mahony L, Fallon PG, Brayden DJ. 2008. Effects of Lactobacillus salivarius 433118 on Intestinal Inflammation, Immunity Status and In vitro Colon Function in Two Mouse Models of Inflammatory Bowel Disease. Dig Dis Sci 53:2495–2506.

4. Tinrat S, Saraya S, Traidej Chomnawang M. 2011. Isolation and characterization of Lactobacillus salivarius MTC 1026 as a potential probiotic. J Gen Appl Microbiol 57:365–378.

5. Gilbert JA, Jansson JK, Knight R. 2014. The Earth Microbiome project: successes and aspirations. BMC Biol 12.

6. Caporaso JG, Lauber CL, Walters WA, Berg-Lyons D, Huntley J, Fierer N, Owens SM, Betley J, Fraser L, Bauer M, Gormley N, Gilbert JA, Smith G, Knight R. 2012. Ultra-high-throughput microbial community analysis on the Illumina HiSeq and MiSeq platforms. ISME J 6:1621–1624.

7. Caporaso JG, Kuczynski J, Stombaugh J, Bittinger K, Bushman FD, Costello EK, Fierer N, Peña AG, Goodrich JK, Gordon JI, Huttley GA, Kelley ST, Knights D, Koenig JE, Ley RE, Lozupone CA, McDonald D, Muegge BD, Pirrung M, Reeder J, Sevinsky JR, Turnbaugh PJ, Walters WA, Widmann J, Yatsunenko T, Zaneveld J, Knight R. 2010. QIIME allows analysis of high-throughput community sequencing data. Nat Methods 7:335–336.

8. Edgar RC, Haas BJ, Clemente JC, Quince C, Knight R. 2011. UCHIME improves sensitivity and speed of chimera detection. Bioinformatics 27:2194–2200.

9. McDonald D, Price MN, Goodrich J, Nawrocki EP, DeSantis TZ, Probst A, Andersen GL, Knight R, Hugenholtz P. 2012. An improved Greengenes taxonomy with explicit ranks for ecological and evolutionary analyses of bacteria and archaea. ISME J 6:610–618.

10. Bik EM, Costello EK, Switzer AD, Callahan BJ, Holmes SP, Wells RS, Carlin KP, Jensen ED, Venn-Watson S, Relman DA. 2016. Marine mammals harbor unique microbiotas shaped by and yet distinct from the sea. Nat Commun 7:10516.

11. Eren AM, Maignien L, Sul WJ, Murphy LG, Grim SL, Morrison HG, Sogin ML, Freckleton R. 2013. Oligotyping: differentiating between closely related microbial taxa using 16S rRNA gene data. Methods Ecol Evol 4:1111–1119.

12. Wickham H. 2011. The Split-Apply-Combine Strategy for Data Analysis. J Stat Softw 40:1–29.

13. Wickham H, Francois R, Henry L, Müller K. 2017. dplyr: A Grammar of Data Manipulation.

14. Wickham H. 2016. tidyr: Easily Tidy Data with `spread()` and `gather()` Functions.

15. Oksanen J, Blanchet FG, Friendly M, Kindt R, Legendre P, McGlinn D, Minchin PR, O’Hara RB, Simpson GL, Solymos P, Stevens MHH, Szoecs E, Wagner H. 2016. vegan: Community Ecology Package.

16. Pohlert T. 2014. The Pairwise Multiple Comparison of Mean Ranks Package (PMCMR).

17. Chen H. 2016. VennDiagram: Generate High-Resolution Venn and Euler Plots.

18. Fellows I. 2012. Deducer: A Data Analysis GUI for R. J Stat Softw 49:1–15.

19. Langfelder P, Horvath S. 2008. WGCNA: an R package for weighted correlation network analysis. BMC Bioinformatics 9:559.

20. Benjamini Y, Krieger AM, Yekutieli D. 2006. Adaptive linear step-up procedures that control the false discovery rate. Biometrika 93:491–507.

21. Pollard KS, Dudoit S, Laan MJ van der. 2005. Multiple Testing Procedures: R multtest Package and Applications to Genomics, in Bioinformatics and Computational Biology Solutions Using R and Bioconductor. Springer.

22. Feizi S, Marbach D, Médard M, Kellis M. 2013. Network deconvolution as a general method to distinguish direct dependencies in networks. Nat Biotechnol 31:726–733.

23. Luo F, Zhong J, Yang Y, Scheuermann RH, Zhou J. 2006. Application of random matrix theory to biological networks. Phys Lett A 357:420–423.

24. Cardona C. 2017. CAVNet: Creation Analysis and Visualization of Networks Package. Bitbucket.

25. Love M, Anders S, Huber W. 2014. Differential analysis of count data–the DESeq2 package. Genome Biol 15:550.

26. Hartemink A, others. Banjo: Bayesian Network Inference with Java Objects (2005).

27. Butts CT. 2016. sna: Tools for Social Network Analysis.
