# Supplementary material for: Environmental Sources of Bacteria Differentially Influence Host-Associated Microbial Dynamics
Source: mSystems. 2018 May 29;3(3):e00052-18. doi: 10.1128/mSystems.00052-18 (PMC5974334; doi:10.1128/mSystems.00052-18)
Supplement: TABLE S1 [file sys003182234st1.docx]

| **Dates\Sources** | **Air** | **Dolphin** | **Food** | **Human** | **Water** | **Total** |
| --- | --- | --- | --- | --- | --- | --- |
| _none | 0 | 2 | 0 | 0 | 0 | 2 |
| 9/24/14 | 0 | 20 | 2 | 16 | 2 | 40 |
| 9/25/14 | 1 | 24 | 2 | 12 | 2 | 41 |
| 9/26/14 | 0 | 22 | 2 | 16 | 2 | 42 |
| 9/27/14 | 3 | 22 | 2 | 16 | 1 | 44 |
| 9/28/14 | 3 | 20 | 2 | 16 | 2 | 43 |
| 9/29/14 | 3 | 141 | 2 | 76 | 11 | 233 |
| 9/30/14 | 2 | 26 | 2 | 14 | 2 | 46 |
| 10/1/14 | 2 | 24 | 0 | 16 | 1 | 43 |
| 10/2/14 | 3 | 24 | 2 | 16 | 2 | 47 |
| 10/3/14 | 3 | 24 | 2 | 14 | 1 | 44 |
| 10/4/14 | 3 | 22 | 2 | 16 | 0 | 43 |
| 10/5/14 | 3 | 20 | 2 | 16 | 2 | 43 |
| 10/6/14 | 3 | 22 | 2 | 16 | 2 | 45 |
| 10/7/14 | 3 | 19 | 2 | 16 | 1 | 41 |
| 10/8/14 | 3 | 18 | 2 | 16 | 2 | 41 |
| 10/9/14 | 1 | 24 | 2 | 16 | 1 | 44 |
| 10/10/14 | 2 | 24 | 2 | 16 | 3 | 47 |
| 10/11/14 | 3 | 24 | 2 | 14 | 2 | 45 |
| 10/12/14 | 3 | 24 | 2 | 16 | 2 | 47 |
| 10/13/14 | 3 | 24 | 2 | 16 | 2 | 47 |
| 10/14/14 | 3 | 94 | 2 | 74 | 11 | 184 |
| 10/15/14 | 2 | 22 | 2 | 16 | 3 | 45 |
| 10/16/14 | 2 | 24 | 2 | 16 | 3 | 47 |
| 10/17/14 | 2 | 24 | 2 | 14 | 3 | 45 |
| 10/18/14 | 2 | 24 | 2 | 16 | 3 | 47 |
| 10/19/14 | 2 | 22 | 2 | 16 | 3 | 45 |
| 10/20/14 | 2 | 24 | 2 | 16 | 2 | 46 |
| 10/21/14 | 2 | 24 | 0 | 16 | 2 | 44 |
| 10/22/14 | 2 | 24 | 2 | 16 | 2 | 46 |
| 10/23/14 | 2 | 24 | 2 | 16 | 2 | 46 |
| 10/24/14 | 2 | 24 | 2 | 16 | 2 | 46 |
| 10/25/14 | 2 | 24 | 2 | 15 | 2 | 45 |
| 10/26/14 | 2 | 24 | 2 | 16 | 2 | 46 |
| 10/27/14 | 1 | 24 | 2 | 16 | 1 | 44 |
| 10/28/14 | 2 | 24 | 2 | 16 | 2 | 46 |
| 10/29/14 | 2 | 23 | 2 | 16 | 2 | 45 |
| 10/30/14 | 2 | 24 | 2 | 16 | 2 | 46 |
| 10/31/14 | 2 | 22 | 2 | 16 | 2 | 44 |
| 11/1/14 | 2 | 24 | 2 | 16 | 2 | 46 |
| 11/2/14 | 2 | 24 | 2 | 16 | 2 | 46 |
| 11/3/14 | 2 | 24 | 2 | 16 | 2 | 46 |
| 11/4/14 | 2 | 116 | 2 | 80 | 7 | 207 |
| Grand Total | 91 | 1253 | 80 | 841 | 105 | **2370** |
