# Supplementary material for: Environmental Sources of Bacteria Differentially Influence Host-Associated Microbial Dynamics
Source: mSystems. 2018 May 29;3(3):e00052-18. doi: 10.1128/mSystems.00052-18 (PMC5974334; doi:10.1128/mSystems.00052-18)
Supplement: TABLE S2 [file sys003182234st2.docx]

| OTUID | Kingdom | Phylum | Class | Order | Family | Genus | Specie | log2FoldChange | pvalue_adjusted | change |
| --- | --- | --- | --- | --- | --- | --- | --- | --- | --- | --- |
| New.0.CleanUp.ReferenceOTU118938 | k__Bacteria | p__Proteobacteria | c__Gammaproteobacteria | o__Pasteurellales | f__Pasteurellaceae | g__Haemophilus | s__parainfluenzae | 5.9 | 0.00062 | Increase |
| New.0.CleanUp.ReferenceOTU1888 | k__Bacteria | p__Firmicutes | c__Clostridia | o__Clostridiales | f__Peptostreptococcaceae | Unknown | Unknown | 7.3 | 0.00001 | Increase |
| New.0.CleanUp.ReferenceOTU30221 | k__Bacteria | p__Proteobacteria | c__Gammaproteobacteria | o__Pasteurellales | f__Pasteurellaceae | Unknown | Unknown | 5.4 | 0.00009 | Increase |
| New.0.CleanUp.ReferenceOTU36315 | k__Bacteria | p__Proteobacteria | c__Gammaproteobacteria | o__Pasteurellales | f__Pasteurellaceae | Unknown | Unknown | 7.1 | 0.00009 | Increase |
| New.0.CleanUp.ReferenceOTU6261 | k__Bacteria | p__Fusobacteria | c__Fusobacteriia | o__Fusobacteriales | f__Fusobacteriaceae | g__Fusobacterium | Unknown | 5.4 | 0.00031 | Increase |
| New.0.CleanUp.ReferenceOTU83346 | k__Bacteria | p__Fusobacteria | c__Fusobacteriia | o__Fusobacteriales | f__Fusobacteriaceae | g__Fusobacterium | Unknown | 6.4 | 0.00009 | Increase |
| New.0.ReferenceOTU43 | k__Bacteria | p__Actinobacteria | c__Actinobacteria | o__Actinomycetales | f__Brevibacteriaceae | g__Brevibacterium | Unknown | -4.6 | 0.00009 | Decrease |
