# Supplementary material for: Environmental Sources of Bacteria Differentially Influence Host-Associated Microbial Dynamics
Source: mSystems. 2018 May 29;3(3):e00052-18. doi: 10.1128/mSystems.00052-18 (PMC5974334; doi:10.1128/mSystems.00052-18)
Supplement: TABLE S3 [file sys003182234st3.docx]

| Location tested | Samples tested | Test | PD Permutation T-Test, *P*-value | PD F-Test, *P*-value |
| --- | --- | --- | --- | --- |
| Dolphin's chuff | Dolphins Group A only | Samples before vs while taking probiotics | 0.168 | 0.000 |
| Dolphin's rectum | Dolphins Group A only | Samples before vs while taking probiotics | 0.049 | 0.000 |
| Dolphin's skin | Dolphins Group A only | Samples before vs while taking probiotics | 0.352 | 0.047 |
| Dolphin's chuff | Dolphins Group B only | Samples before vs while taking probiotics | 0.111 | 0.000 |
| Dolphin's rectum | Dolphins Group B only | Samples before vs while taking probiotics | 0.653 | 0.019 |
| Dolphin's skin | Dolphins Group B only | Samples before vs while taking probiotics | 0.279 | 0.047 |
| Air | All | Samples before vs while taking probiotics | 0.001 | 0.186 |
| Food | All | Samples before vs while taking probiotics | 0.428 | 0.108 |
| Human hand | All | Samples before vs while taking probiotics | 0.999 | 0.000 |
| Human nose | All | Samples before vs while taking probiotics | 0.663 | 0.150 |
| Water | All | Samples before vs while taking probiotics | 0.478 | 0.950 |
| Dolphin's chuff | All before taking probiotics | Group A vs Group B | 0.841 | 0.894 |
| Dolphin's rectum | All before taking probiotics | Group A vs Group B | 0.050 | 0.021 |
| Dolphin's skin | All before taking probiotics | Group A vs Group B | 0.959 | 0.450 |
| Dolphin's chuff | All while taking probiotics | Group A vs Group B | 0.913 | 0.455 |
| Dolphin's rectum | All while taking probiotics | Group A vs Group B | 0.177 | 0.070 |
| Dolphin's skin | All while taking probiotics | Group A vs Group B | 0.794 | 0.385 |
