# Supplementary material for: Environmental Sources of Bacteria Differentially Influence Host-Associated Microbial Dynamics
Source: mSystems. 2018 May 29;3(3):e00052-18. doi: 10.1128/mSystems.00052-18 (PMC5974334; doi:10.1128/mSystems.00052-18)
Supplement: TABLE S4 [file sys003182234st4.docx]

| Distance  Type | Location Tested | Samples  Tested | Test  Type | Anosim R-value | Anosim  *P*-value |
| --- | --- | --- | --- | --- | --- |
| WU | Dolphin's chuff | Dolphins Group A only | Samples before vs while taking probiotics | 0.007 | 0.275 |
| WU | Dolphin's rectum | Dolphins Group A only | Samples before vs while taking probiotics | 0.122 | 0.001 |
| WU | Dolphin's skin | Dolphins Group A only | Samples before vs while taking probiotics | 0.109 | 0.001 |
| WU | Dolphin's chuff | Dolphins Group B only | Samples before vs while taking probiotics | 0.010 | 0.271 |
| WU | Dolphin's rectum | Dolphins Group B only | Samples before vs while taking probiotics | 0.081 | 0.011 |
| WU | Dolphin's skin | Dolphins Group B only | Samples before vs while taking probiotics | 0.082 | 0.004 |
| WU | Air | All | Samples before vs while taking probiotics | 0.080 | 0.014 |
| WU | Food | All | Samples before vs while taking probiotics | -0.006 | 0.547 |
| WU | Human's hand | All | Samples before vs while taking probiotics | 0.057 | 0.003 |
| WU | Human's nose | All | Samples before vs while taking probiotics | 0.055 | 0.003 |
| WU | Water | All | Samples before vs while taking probiotics | 0.009 | 0.347 |
| WU | Dolphin's chuff | All before taking probiotics | Group A vs Group B | 0.486 | 0.001 |
| WU | Dolphin's rectum | All before taking probiotics | Group A vs Group B | 0.107 | 0.001 |
| WU | Dolphin's skin | All before taking probiotics | Group A vs Group B | 0.018 | 0.112 |
| WU | Dolphin's chuff | All while taking probiotics | Group A vs Group B | 0.386 | 0.001 |
| WU | Dolphin's rectum | All while taking probiotics | Group A vs Group B | 0.193 | 0.001 |
| WU | Dolphin's skin | All while taking probiotics | Group A vs Group B | 0.046 | 0.015 |
| UWU | Dolphin's chuff | Dolphins Group A only | Samples before vs while taking probiotics | 0.135 | 0.001 |
| UWU | Dolphin's rectum | Dolphins Group A only | Samples before vs while taking probiotics | 0.268 | 0.001 |
| UWU | Dolphin's skin | Dolphins Group A only | Samples before vs while taking probiotics | 0.273 | 0.001 |
| UWU | Dolphin's chuff | Dolphins Group B only | Samples before vs while taking probiotics | 0.215 | 0.001 |
| UWU | Dolphin's rectum | Dolphins Group B only | Samples before vs while taking probiotics | 0.185 | 0.001 |
| UWU | Dolphin's skin | Dolphins Group B only | Samples before vs while taking probiotics | 0.161 | 0.001 |
| UWU | Air | All | Samples before vs while taking probiotics | 0.114 | 0.006 |
| UWU | Food | All | Samples before vs while taking probiotics | 0.028 | 0.207 |
| UWU | Human's hand | All | Samples before vs while taking probiotics | 0.107 | 0.001 |
| UWU | Human's nose | All | Samples before vs while taking probiotics | 0.119 | 0.001 |
| UWU | Water | All | Samples before vs while taking probiotics | 0.017 | 0.266 |
| UWU | Dolphin's chuff | All before taking probiotics | Group A vs Group B | 0.238 | 0.001 |
| UWU | Dolphin's rectum | All before taking probiotics | Group A vs Group B | 0.164 | 0.001 |
| UWU | Dolphin's skin | All before taking probiotics | Group A vs Group B | 0.017 | 0.095 |
| UWU | Dolphin's chuff | All while taking probiotics | Group A vs Group B | 0.329 | 0.001 |
| UWU | Dolphin's rectum | All while taking probiotics | Group A vs Group B | 0.258 | 0.001 |
| UWU | Dolphin's skin | All while taking probiotics | Group A vs Group B | 0.030 | 0.028 |
