# Supplementary material for: Environmental Sources of Bacteria Differentially Influence Host-Associated Microbial Dynamics
Source: mSystems. 2018 May 29;3(3):e00052-18. doi: 10.1128/mSystems.00052-18 (PMC5974334; doi:10.1128/mSystems.00052-18)
Supplement: TABLE S5 [file sys003182234st5.docx]

|  | A rectum | A Skin | A Chuf | B rectum | B Skin | B Chuf | Water | Food | Air | Human nose | Human hand |
| --- | --- | --- | --- | --- | --- | --- | --- | --- | --- | --- | --- |
| Module_01 | NS | Down | Down | Down | Down | Down | NS | NS | Down | NS | NS |
| Module_02 | NS | NS | NS | NS | NS | NS | NS | NS | NS | Down | NS |
| Module_03 | NS | Down | NS | NS | NS | NS | NS | NS | NS | NS | NS |
| Module_06 | Up | Up | Up | Up | Up | Up | NS | NS | Up | NS | NS |
| Module_07 | Down | Down | Down | Down | Down | Down | NS | NS | NS | Down | Down |
