# Supplementary material for: Environmental Sources of Bacteria Differentially Influence Host-Associated Microbial Dynamics
Source: mSystems. 2018 May 29;3(3):e00052-18. doi: 10.1128/mSystems.00052-18 (PMC5974334; doi:10.1128/mSystems.00052-18)
Supplement: TABLE S6 [file sys003182234st6.docx]

| **Sample Type** | **MMP** | **Shedd** | **Wild** | **Grand Total** |
| --- | --- | --- | --- | --- |
| Air |  | 67 |  | 67 |
| Dol_Blwh | 22 |  |  | 22 |
| Dol_Chuf | 16 | 202 |  | 218 |
| Dol_Gast | 39 |  |  | 39 |
| Dol_Oral | 6 |  | 8 | 14 |
| Dol_Rect | 32 | 201 | 7 | 240 |
| Dol_Skin |  | 204 |  | 204 |
| Fish_&_Squid(Food) | 4 | 39 |  | 43 |
| Human_Hand |  | 208 |  | 208 |
| Human_Nose |  | 209 |  | 209 |
| Sln_Gast | 11 |  |  | 11 |
| Sln_Oral | 4 |  |  | 4 |
| Sln_Rect | 16 |  |  | 16 |
| Water | 28 | 84 | 8 | 120 |
| Grand Total | 178 | 1214 | 23 | 1415 |
