# Supplementary material for: Environmental Sources of Bacteria Differentially Influence Host-Associated Microbial Dynamics
Source: mSystems. 2018 May 29;3(3):e00052-18. doi: 10.1128/mSystems.00052-18 (PMC5974334; doi:10.1128/mSystems.00052-18)
Supplement: FIG S1 [file sys003182234sf1.pdf]

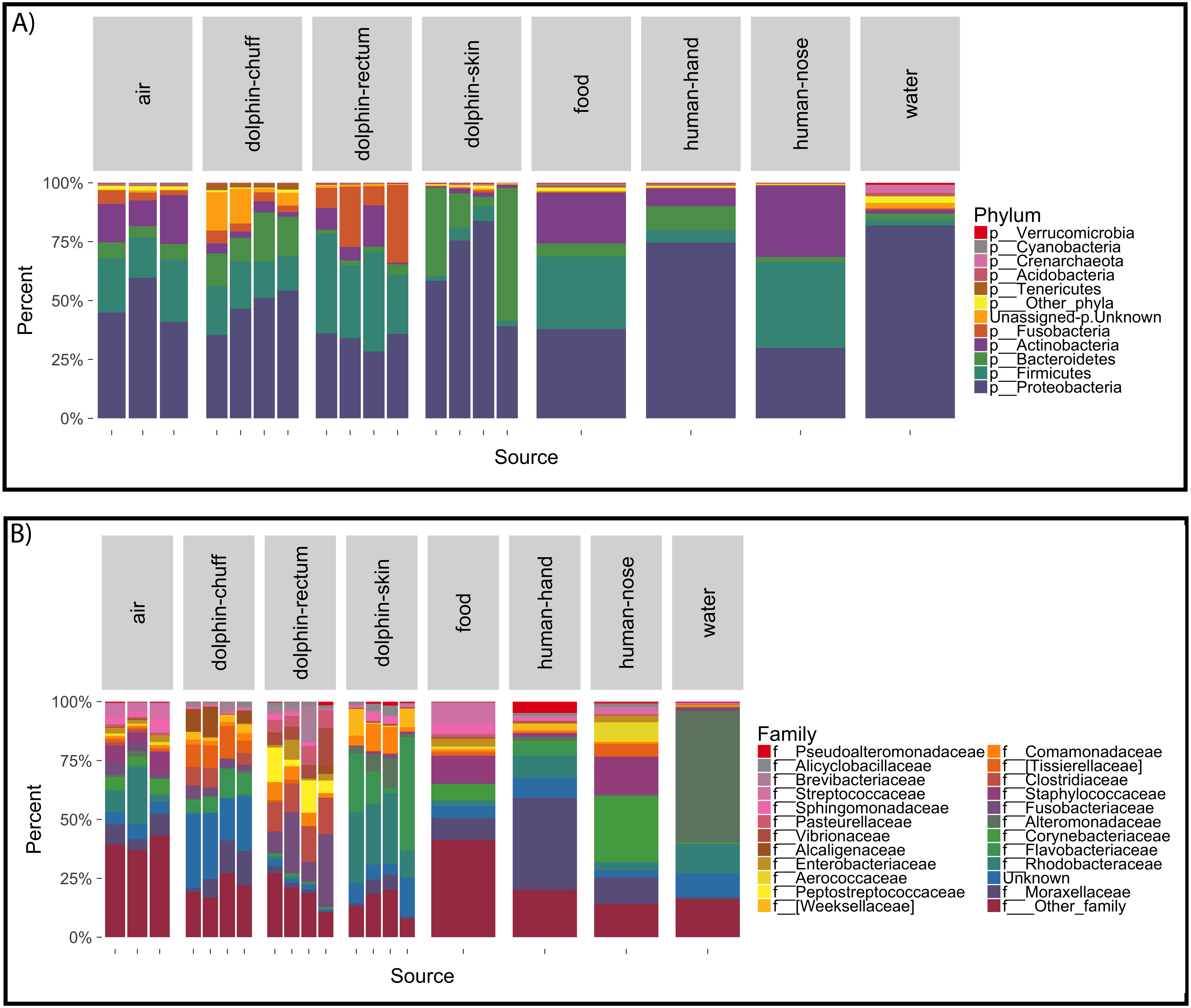
A)
air
dolphin-chuff
dolphin-rectum
dolphin-skin
food
human-hand
human-nose
water
100%
75%
50%
25%
0%
Percent
Source
Phylum
p__Verrucomicrobia
p__Cyanobacteria
p__Crenarchaeota
p__Acidobacteria
p__Tenericutes
p___Other_phyla
Unassigned-p.Unknown
p__Fusobacteria
p__Actinobacteria
p__Bacteroidetes
p__Firmicutes
p__Proteobacteria
B)
Family
f__Pseudoalteromonadaceae
f__Alicyclobacillaceae
f__Brevibacteriaceae
f__Streptococcaceae
f__Sphingomonadaceae
f__Pasteurellaceae
f__Vibrionaceae
f__Alcaligenaceae
f__Enterobacteriaceae
f__Aerococcaceae
f__Peptostreptococcaceae
f__[Weeksellaceae]
f__Comamonadaceae
f__[Tissierellaceae]
f__Clostridiaceae
f__Staphylococcaceae
f__Fusobacteriaceae
f__Alteromonadaceae
f__Corynebacteriaceae
f__Flavobacteriaceae
f__Rhodobacteraceae
Unknown
f__Moraxellaceae
f___Other_family
